# Annotations

**Published:** 1911-05-06

**Authors:** 


### Annotations.

### "Phossy-jaw" in the States.

Some time ago attention was drawn in this Journal to the prevalence of phosphorus poisoning among match-makers in the United States. "Phossy-jaw" has been more prevalent in that than in any other civilised country in the world. In England it is now a pathological curiosity, though on the Continent, particularly in Austria and Italy, where ordinary phosphorus is largely used in the manufacture of matches, it is still relatively common. In America there are peculiar circumstances which have so far led to the retention of the older methods employed in match-making. Owing to the Diamond Match Company, popularly known as the "Match Trust," holding patents for the manufacture of matches by the sesquisulphide or red phosphorus method, other firms were obliged to retain the old yellow phosphorus method in the manufacture of their goods. It is pleasing to be able to state that the way is now clear for Congress to enact legislation dealing with phosphorus poisoning in match factories. The Diamond Match Company, at the personal appeal of President Taft, has formally renounced its claims and dedicated its patents to the people of the United States for their free use. Thus the way is clear for the elimination for ever of one of the most revolting of diseases connected with occupation, and the United States, after lagging behind for fully a quarter of a century, has at last come into line with the rest of the world in dealing with a readily preventible evil.

### Workmen's Accidents.

According to certain figures recently published by the French Ministry of Labour, the number of accidents arising out of employment and giving rise to incapacity lasting over four days was 389,249 in 1910, or an increase of 8 per cent. over the figures of 1909. This increase is noted in all forms of labour with the exception of cutters of precious stones and bakers. The industry in which the greatest number of accidents was reported was that of metallurgy, a proportion of 277 accidents for each 1,000 men so employed. Stone workers had 156 accidents per 1,000 employed, ordinary metal workers 149 per 1,000, and labourers in the chemical industry 143 per 1,000. English statistics, so far as they are available, support these figures. The dangerous trades, so far as accidents to ordinary working-men are concerned, must, therefore, be considered those in which—as in metallurgy, for example—ordinary labourers are put to certain work, which, although not skilled labour in the strict definition of the term, is yet of a nature to demand special aptitude and a certain amount of training. How far the law of cause and effect rules in these accidents is not as yet fully known. There are many points of great interest connected with what may be called the physiology of certain skilled and unskilled trades, and it is time that science devoted some consideration to these points. As we have already stated, we consider it imperatively desirable that medical men should pay more attention to the study of the problems of labour than has hitherto been done.

### A Practitioner's Reference Book.

The number of new pharmacological compounds, synthetic and otherwise, is so large and their indications are so diverse that the average practitioner certainly needs a guide through the long list. A very successful attempt to furnish such a handy reference book to the newer remedies is the annual list issued by the J. D. Riedel Company, of Berlin, under the title of "Riedel's Berichte und Mentor." This gives full details, with dosages, of all the new remedies, together with references to the literature. The notes are arranged in alphabetical order and any given new remedy is therefore easy to find. A highly interesting and useful addendum is the table of patent medicines, giving their composition. A glance through the pages of this list shows very clearly how amazingly fertile of synthetic remedies pharmacological laboratories have been during the past year, and at the same time it gives some indication of the hardship chemists are put to, presumably, to find names for their new compounds! When a new drug has to be designated by a collection of twenty-seven letters, arranged in an order which severely taxes the pronouncing powers of even those of us who have learned the intricacies of the older chemical nomenclature, it is easy to see why numerical titles are becoming popular. The list is forwarded free on the application of any practitioner to the London offices of the firm, 54 Cannon Street. It is in German, but we hope that Messrs. Riedel will consider the desirability of issuing an English edition of it in the future.

### Women and Drink.

The Liverpool Licensing Justices are proposing to issue special regulations with regard to the serving of women in public-houses, of which the most stringent are that any woman whose appearance is not respectable should be refused altogether, and that when women are served they shall only be served once, and shall not be allowed to treat each other to drinks. This "differential legislation," as the *Charity Organisation Review* calls it, verges well-nigh on the intolerable, and can only be the product of narrow-minded fanaticism on the part of the teetotal brigade. That disreputable persons of both sexes should be deprived of further liquor when in a state of intoxication is not only right but imperative; but that perfectly decent members of the female sex should be dictated to as to the number of glasses of alcoholic refreshment they may partake of, as well as to the sex of the person to whom they may offer hospitality (for, as the *Review* points out, nothing is said of women treating men, nor of men treating women) amounts to almost a breach of the liberties of the subject, and the mere proposal of such specious legislation in these days of female suffragism can only tend to heap ridicule on the heads of its fantastic originators. Licensing Justices should remember that their very name indicates that they are appointed to administer justice and not to air their own views on a particular subject.

